# Three-Dimensional Evaluation Effects of Microimplant-Assisted Rapid Palatal Expansion on the Upper Airway Volume: A Systematic Review and Meta-Analysis

**DOI:** 10.3390/jcm12051790

**Published:** 2023-02-23

**Authors:** Lan Li, Mingrui Zhai, Mengqiao Wang, Shuyue Cui, Chen Cheng, Jixiao Wang, Fulan Wei

**Affiliations:** 1 Department of Orthodontics, School and Hospital of Stomatology, Cheeloo College of Medicine, Shandong University, Jinan 250012, China; 2 Shandong Key Laboratory of Oral Tissue Regeneration & Shandong Engineering Laboratory for Dental Materials and Oral Tissue Regeneration, Jinan 250012, China

**Keywords:** upper airway, palatal expansion technique, systematic review, meta-analysis

## Abstract

Microimplant-assisted rapid palatal expansion is increasingly used clinically; however, the effect on the upper airway volume in patients with maxillary transverse deficiency has not been thoroughly evaluated yet. The following electronic databases were searched up to August 2022: Medline via Ovid, Scopus, Embase, Web of Science, Cochrane Library, Google Scholar, and ProQuest. The reference lists of related articles were also reviewed by manual search. The Revised Cochrane Risk of Bias Tool for randomized trials (ROB2) and the Risk of Bias in non-randomized Studies of Interventions (ROBINS-I) tool were used to evaluate the risks of bias of the included studies. The mean differences (MD) and 95% confidence intervals (CI) of changes in nasal cavity and upper airway volume were analyzed using a random-effects model, and subgroup and sensitivity analyses were also performed. Two reviewers independently completed the process of screening studies, extracting data, and assessing the quality of studies. In total, twenty-one studies met the inclusion criteria. After assessing the full texts, only thirteen studies were included, with nine studies selected for quantitative synthesis. Oropharynx volume increased significantly after immediate expansion (WMD: 3156.84; 95% CI: 83.63, 6230.06); however, there was no significant change in nasal volume (WMD: 2527.23; 95% CI: −92.53, 5147.00) and nasopharynx volume (WMD: 1138.29; 95% CI: −52.04, 2328.61). After retention a period, significant increases were found in nasal volume (WMD: 3646.27; 95% CI: 1082.77, 6209.77) and nasopharynx volume (WMD: 1021.10; 95% CI: 597.11, 1445.08). However, there was no significant change after retention in oropharynx volume (WMD: 789.26; 95% CI: −171.25, 1749.76), palatopharynx volume (WMD: 795.13; 95% CI: −583.97, 2174.22), glossopharynx volume (WMD: 184.50; 95% CI: −1745.97, 2114.96), and hypopharynx volume (WMD: 39.85; 95% CI: −809.77, 889.46). MARPE appears to be linked with long-term increases in nasal and nasopharyngeal volume. However, high-quality clinical trials are required to further verify the effects of MARPE treatment on the upper airway.

## 1. Introduction

Maxillary transverse deficiency is a common malocclusion characterized by dental crowding, narrow nasal cavity, high palatal vault, and unilateral or bilateral posterior crossbite [1,2]. Compared with normal individuals, patients with maxillary constriction often have narrow airways [3]. It is known that the decrease in the amount of air passing through the nasal cavity into the lungs can affect craniofacial growth and development as well as overall health [4]. Moreover, some studies have reported that maxillary transverse deficiency can be a possible cause of obstructive sleep apnea (OSA) [5,6].

Rapid palatal expansion (RPE) is often used to correct the narrow maxilla by separating the midpalatal suture. However, because the skeletal resistance of the midpalatal suture gradually increases with age [7] and some side effects may occur after RPE treatment, such as anchored teeth root resorption [8], dehiscence, and fenestration of the buccal cortex [9], traditional rapid palatal expansion in mature patients is still questionable. Surgically-assisted rapid palatal expansion (SARPE) has been proposed to achieve skeletal expansion in mature patients with transverse maxillary deficiencies [10]. However, complications and the high cost of surgery disincline some patients from choosing this treatment [11]. Microimplant-assisted rapid palatal expansion (MARPE) was suggested as an alternative to SARPE [12]. Orthodontic micro-implants serve as a skeletal anchorage for RPE, which not only produce more skeletal expansion while reducing adverse dentoalveolar effects, but also can reduce surgical injury [13].

At present, there are many meta-analyses and systematic reviews on the two-dimensional width and three-dimensional volume changes of the upper airway after rapid palatal expansion [14,15,16,17,18,19]. However, only two studies synthesize on the changes of upper airway dimension in MARPE. Krüsi et al. [20] only described the width of the nasal cavity after MARPE. Abu Arqub et al. [21] included only three studies to describe the short-term changes of the upper airway dimension after MARPE but without quantitative analysis, which did not provide solid evidence of the relationship between airway changes and MARPE treatment. Hence, a thorough systematic evaluation of the clinical evidence related to the short-term and long-term changes of the upper airway volume after MARPE is needed to better understand the effects of MARPE on the dimension and function of the upper airway and to determine if the therapy is helpful for patients with airway stenosis.

## 2. Materials and Methods

### 2.1. Protocol and Registration

We used the Cochrane Handbook to perform the review [22], and the PRISMA (Preferred Reporting Items for Systematic reviews and Meta-Analyses) guideline to report our results [23]. The protocol was registered on the PROSPERO database (number CRD42020198286).

### 2.2. Eligibility Criteria

We developed the following inclusion criteria using the principles of population, intervention, comparison, outcome, and study design (PICOS): Participants (P): patients with narrow maxilla needed maxillary expansion treatment. We have no age restrictions for patients who are included. Intervention (I): micro-implant-assisted rapid palatal expansion. Comparison (C): age-and sex-matched patients treated with RPE or SARPE, patients without maxillary expansion treatment, or comparison of the same patients before and after MARPE. Outcome (O): changes in upper airway volume, assessed by CT or CBCT. Study design (S): randomized controlled trials or non-randomized studies. The exclusion criteria included: (1) case reports, animal studies, and reviews. (2) Studies that applied another treatment or auxiliary surgery during micro-implant-assisted rapid palatal expansion. (3) Studies that included patients with craniofacial abnormalities (cleft lip and /or palate). (4) Studies without using CBCT or CT to measure the upper airway volume.

### 2.3. Information Sources and Search Strategy

The published literature was searched in the electronic databases Medline via Ovid, Cochrane Library, Embase, Web of Science, and Scopus, and the databases were searched up to August 2022.The reference lists of related articles were also reviewed to find any probable articles that may be missed during the electronic database searches. No restrictions on the year of publication or language. For grey literature, Google Scholar and ProQuest were searched. The search was performed independently by two authors. The search strategy for each database was shown in Table S1.

### 2.4. Study Selection

Two authors performed the selection process independently. The authors reviewed the titles and abstracts of retrieved articles. When no abstract was available or the abstract do not contain sufficient information, the full text of articles that met the inclusion criteria would be obtained to review. Authors would be contacted if additional information was needed. The reference lists of related studies were also reviewed in the same way to find other articles that met the eligibility criteria. Any discrepancy between the two authors was settled by discussion with a third author.

### 2.5. Data Items and Collection

Two authors used the data extraction form to extract the data independently. Details of the included studies were collected, including authors, year of publication, and study design. Information about the study samples, including the number of participants, gender, age, sample inclusion criteria, control group setting, as well as measure method of the upper airway volume, were recorded. Additionally, the authors retrieved the type of expander and expansion protocol, timing, and retention details from all included studies, and upper airway assessment methods, parameters used for CBCT or CT, and software used for image reconstruction also were recorded. The follow-up points were defined as: T0, before expansion; T1, immediately after expansion; T2, three months retention after expansion; and T3, six months retention after expansion. The volumetric changes in any region of the upper airway and upper airway boundary used in studies were recorded. Anatomically, the upper airway is divided into nasal cavity, paranasal sinuses, nasopharynx, oropharynx, and hypopharynx [24]. 

### 2.6. Risk of Bias in Individual Studies

Two authors evaluated the risk of bias for randomized studies using the Revised Cochrane Risk of Bias (ROB2) tool [25]. For non-randomized studies, the Risk of Bias in Non-randomized Studies—of Interventions (ROBINS-I) tool [26] was used to assess the quality of the included studies. Any disagreements between the authors were resolved through conversation with a third author.

### 2.7. Summary Measures and Approach to Synthesis

Changes in upper airway volume in each segment were regarded as the primary outcome. This systematic review only included studies measuring the upper airway volume with CBCT or CT, which minimized the differences among different studies. Stata MP 16.0 (Stata Corp, College Station, TX, USA) software was used for statistical analysis. All the indicators included in this meta-analysis were continuous variables. The mean differences (MD) and the associated 95% confidence intervals (CI) were calculated for all meta-analyses. 

The heterogeneity test was conducted on the effect values of independent studies. If the heterogeneity was significant, the random effects model was used to combine the effect value according to DerSimonian and Laird [27]. Subgroup analysis was used in the meta-analysis to identify possible sources of heterogeneity. Sensitivity analyses were planned for the meta-analyses to assess their robustness. Egger’s test would be used to assess publication bias if enough trials were included in this meta-analysis. A two-tailed value *p* = 0.05 was considered significant for hypothesis testing, but a two-tailed value *p* = 0.10 was used for heterogeneity testing and publication bias [28]. 

### 2.8. Risk of Bias Assessment across Studies

The overall quality of evidence was assessed using the Grading of Recommendations, Assessment, Development and Evaluation (GRADE) [29]. Two authors performed this analysis independently. Disagreements are resolved in discussion with the third author.

## 3. Results

### 3.1. Study Selection

**Figure 1:**
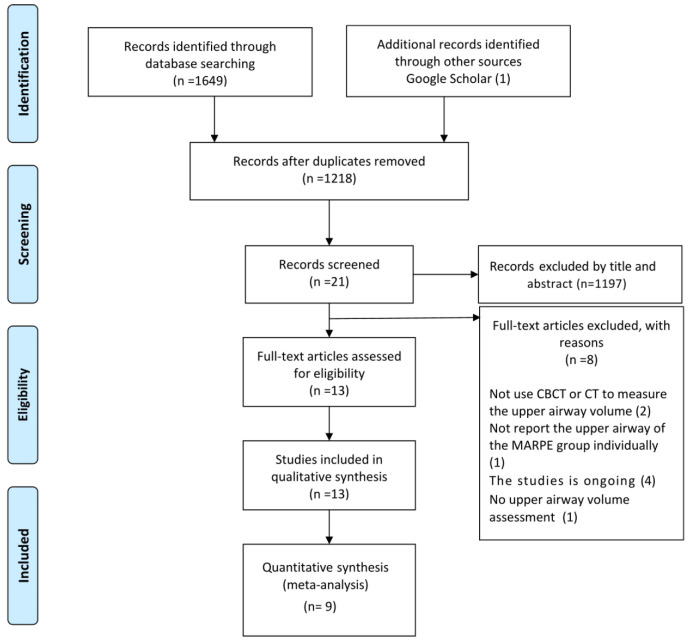
The flow diagram of the study selection process.

In total, 1649 articles were initially identified by means of searching databases (Table S2), and one additional study was retrieved through other sources. In total, 1218 articles were evaluated after excluding duplicates. By reviewing titles and abstracts, 1197 articles were excluded. Thus, the remaining 21 articles were eligible for the full-text assessment. Eight articles were excluded with reasons after reviewing the full texts (Table S3), and 13 articles were eventually included for the qualitative analysis. From these, a total of nine studies were included in the quantitative synthesis, as four studies reported incomplete data. The flow diagram of the study selection process is presented in Figure 1.

### 3.2. Study Characteristics

Table 1, Table 2, Table 3, Tables S4 and S5 present the main information from the included studies in the present systematic review. In terms of study design, three studies were prospective clinical studies [30,31,32] and nine studies [33,34,35,36,37,38,39,40,41] were retrospective studies. The remaining was a randomized controlled trial [42]. All studies had a total of 244 participants, 117 women and 61 men. One study only included male patients [31] and three did not report a male-female ratio [32,38,39]. Patients ranged in age from 8 to 35 years, although one study did not report any age information [41]. Six studies had control groups [31,36,37,38,41,42], but all of them compared different expanders. For the inclusion criteria, all patients had maxillary transverse deficiency, and the inclusion criteria of one study [32] was OSA patients who had obstructive sleep apnea with maxillary transverse deficiency. A maxillary skeletal expander with two micro-implants on each side in a mid-palatal suture to provide support was applied in seven studies [30,32,35,36,39,40,41]. One study applied the palatal bracket implant anchorage arch expander [34]. One study used modified conventional four-banded hyrax expander [33]. Two studies used the bone-borne rapid maxillary expander with one micro-implant on each side of the posterior dental region in palatine to provide support [37,38], and two studies used the hybrid hyrax expander with one implant on each side of the anterior dental region in palatine to provide support [31,42]. All studies reported the expansion protocol; however, the expansion protocol varied. The most common is 0.5 mm/day. Other studies applied 0.2 mm [33], 0.64 mm [36], or 0.25 mm [32] per day, and 0.26 mm every other day [35]. Five studies reported only the extent or amount of eventual expansion without specifying expansion protocol [31,38,39,40,41]. CT images were used to measure upper airway volume in only one of the studies [31], and CBCT was used in the rest. The scanning parameter settings used in the included studies were different, but they all included FOV, voxel size, scan time, KV, and mA. Software packages for 3D image reconstruction included Dolphin software [34,35,37,38,39,40,42], Mimics software [31,32], Invivo software [32], OnDemand3D [30,33,36], and Amira software [41]. All studies recorded data measurements at time points. Changes in the upper airway volume were measured in seven studies immediately after expansion (T1) [30,31,33,36,39,41], four studies at three months after expansion (T2) [34,35,37,40], and two studies at six months after expansion (T3) [32,38,42]. The included studies measured volumetric changes in any region of the upper airway, including the nasal cavity, nasopharynx, oropharynx, hypopharynx, and maxillary sinus. However, the delineation of upper airway boundaries varied among studies. 

### 3.3. Risk of Bias in Individual Studies and across the Studies

**Figure 2:**
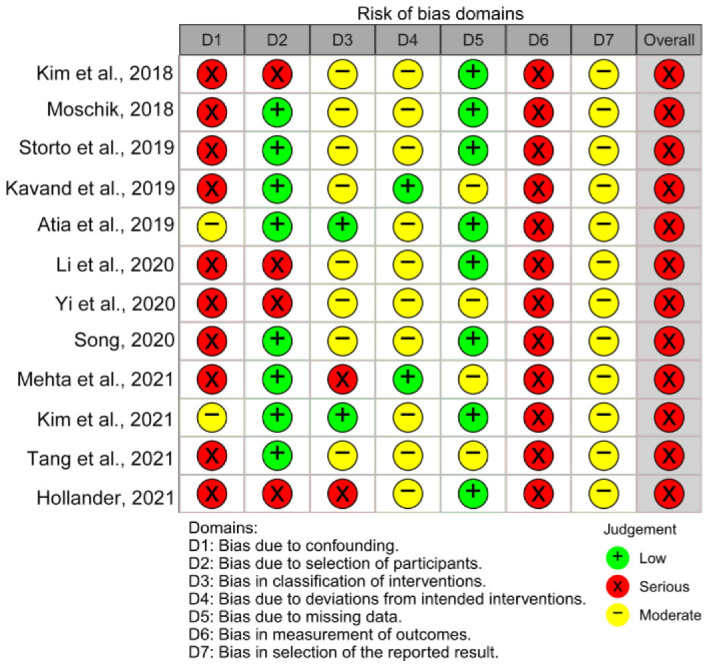
Results of the risk of bias assessment in the individual studies with the Risk of Bias in Non-randomized Studies—of Interventions (ROBINS)-I tool [26,30,31,32,33,34,35,36,37,38,39,40,41].

**Figure 3:**
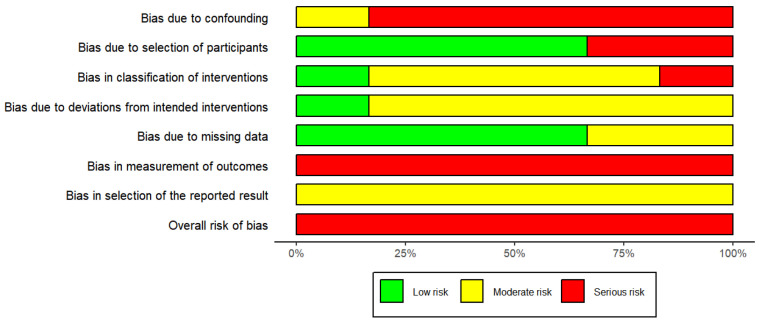
Risk of bias percentage per domain of all included studies assessed with the Risk of Bias in Non-randomized Studies—of Interventions (ROBINS-I) tool.

The risk of bias assessment results for randomized and non-randomized studies were shown in Figure 2, Figure 3 and Figure S1. In total, there were 13 studies, of which was a randomized controlled trial [42] and the other 12 were non-randomized studies [30,31,32,33,34,35,36,37,38,39,40,41]. The ROBINS-I tool was used to assess the 12 non-randomized studies. The outcome of the assessment revealed a serious risk of bias, mostly due to bias in confounding, participant selection, classification of the intervention, and measurement of outcomes. The randomized controlled trial was assessed using ROB2 tool and the result showed some concerns. Of the five assessment domains, the domain in bias due to randomization showed some concerns, and the remaining four domains showed low risk of bias. The GRADE rating of the quality of evidence for the meta-analysis was presented in Table S6. For quality rating, nasal cavity volume after retention were low, and the rest of outcomes was moderate. The main reason for lowering the quality of the evidence was with serious risk of bias, and inconsistency also occurred in some outcomes.

### 3.4. Results of Individual Studies, Meta-Analyses, and Subgroup Analyses

#### 3.4.1. Nasal Cavity Volume

**Figure 4:**
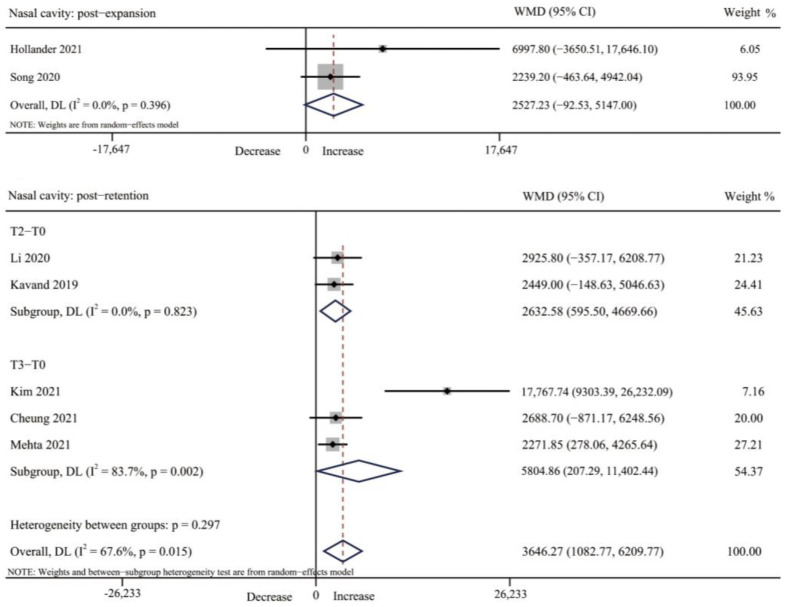
Forest plot for the changes of nasal cavity volume immediately after expansion (T1) and retention (T2–T3). T0, before expansion; T1, immediately after expansion; T2, three months after expansion; T3, six months after expansion; WMD, weighted mean difference; CI, confidence interval [32,35,37,38,39,41,42].

Four studies reported on the changes of nasal volume after expansion [33,36,39,41]. Five studies reported the changes after retention [32,35,37,38,42]. Percentage increases in nasal volume ranged from 9.21% to 22.73% after expansion and 10.1% to 77.2% after retention, and measurements in all studies were statistically significant. However, only two [39,41] and five studies [32,35,37,38,42] on short-term and long-term changes in nasal volume were available for a meta-analysis, respectively. The results showed that there was no change immediately after expansion (WMD = 2527.23 mm^3^, 95% CI: −92.53, 5147.00, *p* = 0.059). However, after a period of retention, nasal volume increased significantly (WMD = 3646.27, 95% CI = 1082.77, 6209.77, *p* = 0.005). For subgroup analysis at different retention time points, nasal volume increased significantly after retention for three months and six months (T2: *p* = 0.011, T3: *p* = 0.042) (Table 3, Figure 4 and Table S7).

#### 3.4.2. Nasopharynx Volume

**Figure 5:**
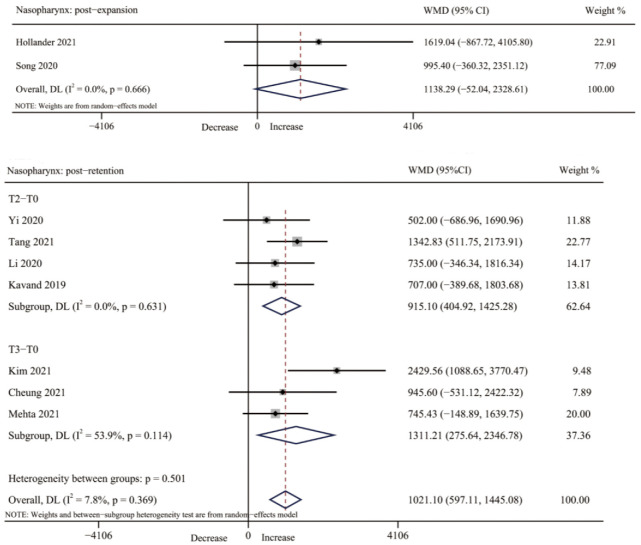
Forest plot for the changes of nasopharynx volume immediately after expansion (T1) and retention (T2–T3). T0, before expansion; T1, immediately after expansion; T2, three months after expansion; T3, six months after expansion; WMD, weighted mean difference; CI, confidence interval [32,34,35,37,38,39,40,41,42].

Four studies [30,33,39,41] assessed changes in nasopharynx volume after expansion, and seven studies [32,34,35,37,38,40,42] after retention. The increase range of nasopharyngeal volume was from 6.4% to 19.99% after expansion and from 8.48% to 47.9% after retention. The changes were statistically significant in all studies except for one study [33]. The pool analysis of two studies [39,41] showed that there was no significant change in nasopharyngeal volume immediately after MARPE treatment (WMD = 1138.29, 95% CI: −52.04, 2328.61, *p* = 0.061). Seven studies [32,34,35,37,38,40,42] were available for a meta-analysis to assess changes in nasopharynx volume after retention, with a statistically significant increase (WMD = 1021.10, 95% CI: 597.11, 1445.08, *p* = 0.000), and subgroup analysis also showed significant changes in nasopharynx volume from T0 to T2 for three months retention as well as from T0 to T3 for six months retention (T2-T0: WMD = 915.10, 95% CI: 404.92, 1425.28, *p* = 0.000. T3-T0: WMD = 1311.21, 95 CI%: 275.64, 2346.78, *p* = 0.013) (Table 3, Figure 5 and Table S7).

#### 3.4.3. Oropharynx Volume

**Figure 6:**
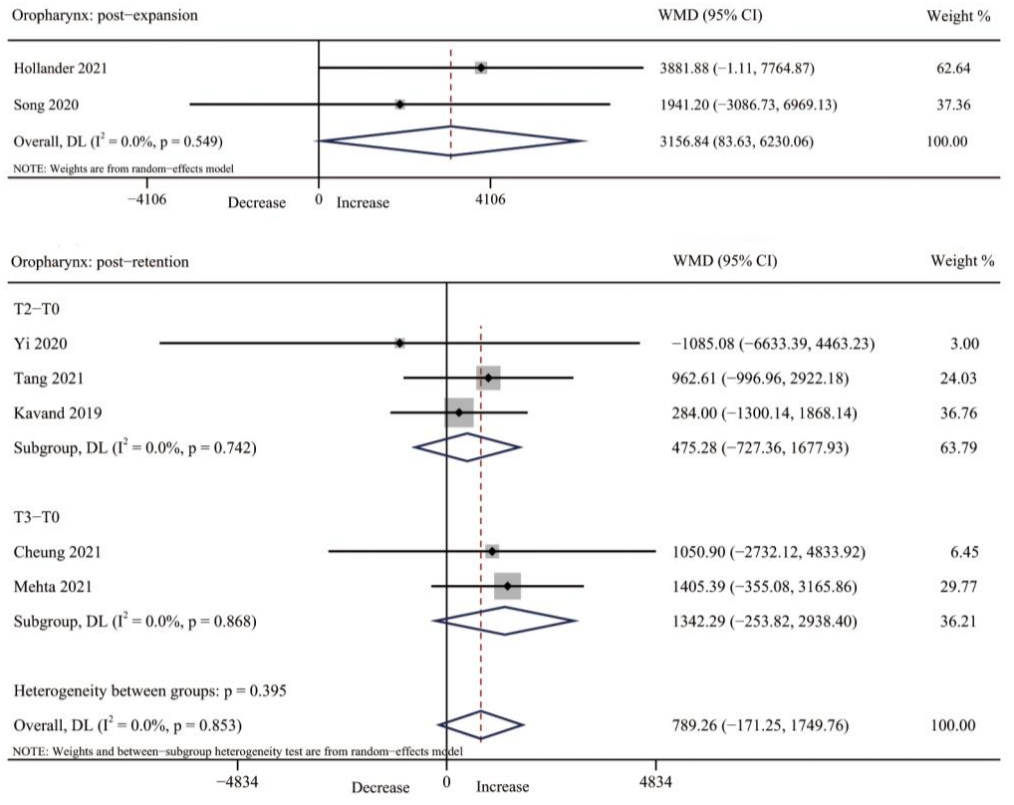
Forest plot for the changes of oropharynx volume immediately after expansion (T1) and retention (T2–T3). T0, before expansion; T1, immediately after expansion; T2, three months after expansion; T3, six months after expansion; WMD, weighted mean difference; CI, confidence interval [34,37,38,39,40,41,42].

The oropharynx volume was reported in three studies after expansion [31,39,41]. However, for the meta-analysis, only two studies [39,41] can be combined, showing the oropharynx volume was significantly increased after expansion (WMD = 3156.84, 95% CI: 83.63, 6230.06, *p* = 0.044), with no significant heterogeneity observed (I^2^ = 0.0%, *p* = 0.549). Five studies [34,37,38,40,42] reported the oropharynx volume after retention. However, there was no significant difference in oropharynx volume after three-month retention and six-month retention (T2–T0: WMD = 475.28, 95% CI: −727.36, 1677.93, *p* = 0.439. T3–T0: WMD = 1342.29, 95% CI: −253.82, 2938.40, *p* = 0.099. Overall WMD = 789.26, 95% CI: −171.25, 1749.76, *p* = 0.107) (Table 3, Figure 6 and Table S7).

#### 3.4.4. Palatopharynx Volume

**Figure 7:**
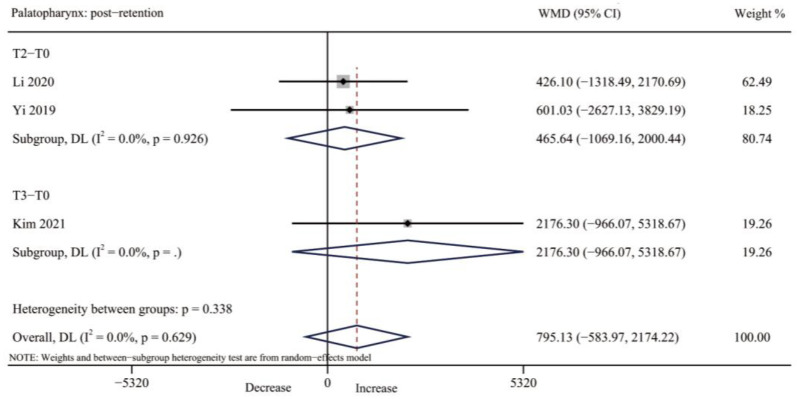
Forest plot for the changes of palatopharynx volume after retention (T2–T3). T0, before expansion; T2, three months after expansion; T3, six months after expansion; WMD, weighted mean difference; CI, confidence interval [32,34,35].

No one study reported the post-expansion changes in palatopharynx volume. Three studies [32,34,35] evaluated the palatopharynx volume after retention and there was no significant increase found (WMD = 795.13, 95% CI: −583.97, 2174.22, *p* = 0.258); however, with a low heterogeneity (I^2^ = 0.0%; *p* = 0.629) (Table 3, Figure 7 and Table S7).

#### 3.4.5. Glossopharynx Volume

**Figure 8:**
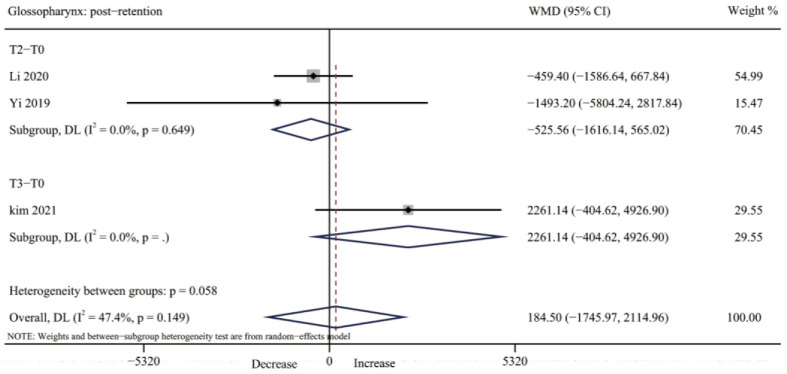
Forest plot for the changes of glossopharynx volume after retention (T2–T3). T0, before expansion; T2, three months after expansion; T3, six months after expansion; WMD, weighted mean difference; CI, confidence interval [32,34,35].

Regarding the glossopharynx volume, no study evaluated glossopharynx volume after expansion. However, three studies [32,34,35] reported the change of the glossopharynx volume after retention; no significant changes were seen (WMD: 184.50; 95% CI: −1745.97, 2114.96, *p* = 0.851) and heterogeneity was moderate (I^2^ = 47.4%, *p* = 0.149) (Table 3, Figure 8 and Table S7).

#### 3.4.6. Hypopharynx Volume

**Figure 9:**
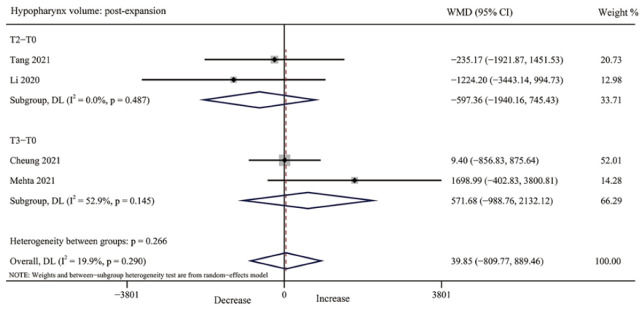
Forest plot for the changes of hypopharynx volume after retention (T2–T3). T0, before expansion; T2, three months after expansion; T3, six months after expansion; WMD, weighted mean difference; CI, confidence interval [35,38,40,42].

For hypopharynx volume, no study was included immediately after MARPE. Four studies [35,38,40,42] reported the volume change after retention, with low to moderate heterogeneity (T2, I^2^ = 0.0%, *p* = 0.487; T3, I^2^ = 52.9%, *p* = 0.145); however, no significant changes were observed in hypopharynx volume (T2, *p* = 0.383, T3, *p* = 0.473, overall *p* = 0.927) (Table 3, Figure 9 and Table S7).

#### 3.4.7. Maxillary Sinus Volume

No study reported the changes of the maxillary sinus volume after expansion. However, the maxillary sinus volume was observed in two studies [37,42], one of which showed the total maxillary sinus volume increased by 10.0% after six months retention [42], the other showed that there was a 2.1% change in the left maxillary sinus volume and 5.2% change in the right maxillary sinus volume (Table 3).

#### 3.4.8. Additional Analyses

Each outcome that included more than three studies was assessed for publication bias, with a total of six outcomes analyzed. According to Egger’s test, no obvious publication bias was found (Table S8). Sensitivity analysis showed that the results were robust after one study was omitted (Table S9). However, due to the small number and the characteristics of the included studies, other sensitivity analyses could not be robustly performed.

## 4. Discussion

### 4.1. Summary of Evidence

Cranial and maxillofacial malformations affecting upper airway volume have been proven to be one of the important causes of obstructive sleep apnea (OSA) [43]. Transverse deficiency of the maxilla is known as a contributor to the development of OSA [44]. Rapid maxillary expansion (RPE) is a common method to correct maxillary transverse deficiency in adolescents. However, in late adolescents and young adults with partial or complete ossification of the mid-palatal suture, MARPE is used to achieve more skeletal expansion [45]. MARPE not only opens the maxillary palatal suture, but also affects the upper airway to varying degrees. Brunetto et al. [46] conducted a study on 16 adult patients with OSA and showed that 6 months after receiving MARPE treatment, the drowsiness, oxygen saturation, and snoring duration were improved. Kim et al. [32] performed micro-implant-assisted palatal expansion on 14 children with OSA and found that MARPE can effectively treat OSA patients. By expanding the nasal and maxillary complex, the airway patency of the nose and pharynx can be enhanced, and pharynx collapse and the nasal airway can be improved eventually. However, due to the lack of research in this area, there are still many controversies about its effect and mechanism on the upper airway. 

Traditionally, the upper airway was measured by 2D imaging (lateral cranial radiographs); however, measuring three-dimensional volume from 2D imaging has considerable limitations [47]. 3D imaging techniques (magnetic resonance imaging, computed tomography, and cone-beam computed tomography) allow airway analysis to be visualized and is more reliable [48,49]. All studies included in this systematic review adopted CBCT or CT 3D imaging technology, which can more intuitively and accurately reflect the changes of upper airway volume after MARPE treatment. Therefore, the purpose of this meta-analysis was to comprehensively analyze the studies on the changes of upper airway volume after micro-implant-assisted rapid palatal expansion for patients with maxillary transverse deficiency by CT and CBCT, so as to provide intentional clinical evidence for clinicians. 

For nasal volume, there was no significant change immediately after expansion, which may be due to only two studies being included to conduct a meta-analysis. Moschik [36] mainly focused on MARPE regarding the movement pattern of the lateral wall of the nasal cavity. Although the study showed that the nasal volume increased after MARPE, it did not report data completely, so it was not included in the quantitative analysis. Significant changes in nasal cavity volume were observed after retention of a period of time, consistent with changes in nasal cavity volume after traditional rapid maxillary expansion [15] and surgically-assisted rapid maxillary expansion [50]. For total effect values, the nasopharyngeal volume also did not change significantly immediately after expansion, which may be related to the small number of studies included. Kim et al. [33] found no significant change in nasopharyngeal volume after MARPE, possibly because the oropharynx was included in Kim’s study.

The oropharynx consists of the glossopharynx and the palatopharynx, so the volume of the oropharynx is closely related to the volume of the palatopharynx and the glossopharynx. Atia et al. [31] reported significant volume changes in the oropharynx compared to other studies. In Atia’s study, all patients were male, and the upper airway space associated with the base of the tongue was subtracted to eliminate the change in airway volume caused by the change in tongue position, which may have resulted in a slight difference. Since some oropharyngeal anatomical structures, including the hyoid bone, tongue, and soft palates, are movable, the possibility of changes in the size and position of these structures due to the influence of gravity should be considered [51]. Tongue position could influence the dimensions of the oropharynx at the time of acquisition of the examination. The lack of control over the soft tissues related to breathing movements and swallowing and tongue positioning can be confounding factors when measuring the oropharynx volume using CBCT or CT, which can cause errors in interpreting the volume of this region. 

Posture is considered to be an important determinant of upper airway size [52,53]. A study comparing changes in upper airway morphology in supine and upright positions found that there was indeed a difference in airway morphology between the two positions: when supine, the airway became significantly smaller, and its resistance increased [54]. In this systematic review, three studies reported supine position [35,39,40,42], two studies [32,34] reported upright position, and the remaining studies did not report patient posture, which may lead to some degree of heterogeneity. On lateral cephalograms, changing head position from natural head position (NHP) to twenty degrees increased the pharyngeal cross-sectional airway dimension and an increase of ten degrees of craniocervical inclination would lead to the pharyngeal airway space accordingly increased by approximately 4 mm [55,56]. Therefore, an alteration in head position can also influence the measurement of upper airway space. Most of the included studies used control measures, such as cranial localization, to minimize the measurement bias. 

Furthermore, sagittal and vertical skeletal pattern can influence the pharyngeal airway dimension. In patients with ANB greater than 4°, the airway was significantly narrower, and the nasopharyngeal and oropharyngeal airway were also negatively correlated with SN-MP angle [57]. Only two studies [35,42] attempted to examine the effect of vertical craniofacial patterns on the upper airway. Different skeletal patterns have different effects on the airway, which can lead to differences between individual outcomes. Further investigations with appropriately grouping patients are needed to more accurately assess upper airway changes after MARPE and provide convincing evidence about this research question. 

### 4.2. Strengths and Limitations

This systematic review used the GRADE approach to assess of the quality of evidence and all steps of qualitative synthesis were performed in accordance with the PRISMA statement [23]. Besides, any literature screening, data extraction, and data analysis was carried out by the two authors and any disagreement was resolved by the third reviewer. All of this increases the accuracy and credibility of the results. Moreover, to our knowledge, this is also the first meta-analysis to comprehensively analyze changes in upper airway volume after MARPE treatment. The limitations of this systematic review are as follows: First, the sample size of quantitative synthesis in the meta-analysis is relatively small. The results may not provide strong evidence of a relationship between airway changes and MARPE treatment. Secondly, the studies included many observational studies, but only one randomized controlled trial, so the quality of the meta-analysis was low. In addition, there were differences in adopting expanders, patient’s position during CBCT or CT photography, the measurement methods and indicators selected, and the retention time after palatal expansion, which may lead to certain clinical heterogeneity among the results of the studies and affect the results of the meta-analysis to some extent. Finally, most existing studies do not have a control group to reduce the confounding effects of normal growth. However, the maximum retention time included in this study was 6 months. If the upper airway of children aged 6–15 increased at a rate of 0.032 cm^3^/ year [58], the effect of growth on upper airway volume would be small. Due to the limitations of this systematic review, the reliability of the meta-analysis results was reduced to some extent. It is suggested to carry out more high-quality, large-sample clinical trials in the future, strictly formulate research plans, unify measurement methods and indicators as far as possible, and obtain more scientific and objective data to provide more high-quality evidence for clinical research.

## 5. Conclusions

MARPE treatment might cause a long-term significant increase in nasal cavity volume and nasopharynx volume, while no significant changes were found in the oropharynx, palatopharynx, glossopharynx, hypopharynx, and maxillary sinus volume. However, given the limited number of existing studies and the problem of different degrees of heterogeneity, these results should be considered with caution. Well-designed and conducted randomized controlled studies are required to further explore this issue.

## Figures and Tables

**Table 1:** Main characteristics of the included studies in this systematic review.

Study	Study Design	Participants	Control	Inclusion Criteria	Intervention	Main Outcome
Storto et al., 2019 [30]	Prospective clinical study	20 pts (13 f, 7 m) mean age: 17.1 yrs	NO	Patients with maxillary transverse deficiency; permanent dentition; CS6 skeletal maturation stage; mouth breathers	Maxillary skeletal expander	Nasopharynx volume Oropharynx volume
Kim et al., 2018 [33]	Retrospective clinical study	14 pts (10 f, 4 m) mean age: 22.76 ± 3.3 yrs range: 18.3–26.5 yrs	NO	Young adults (>18 years of age) with a transverse discrepancy; successful opening of the mid-palatal suture; non-extraction treatment; availability of CBCT images obtained before and after expansion.	Modified conventional four-banded hyrax expander	Nasal cavity volume Nasopharynx volume
Yi et al., 2020 [34]	Retrospective clinical study	13 pts (10 f, 3 m) mean age: 19.95 ± 4.39 yrs range: 15–29 yrs	NO	Maxillary constriction; good oral hygiene and periodontal condition; no history of orthodontics; maxillofacial trauma or respiratory tract therapy; no systemic diseases; no other maxillofacial deformity; did not take long-term drugs; the mid-palatal suture stage was C, D, E; successful maxillary expansion; had follow-up imaging data.	The palatal bracket implant anchorage arch expander	Nasopharynx volume Oropharynx volume Palatopharynx volume Glossopharynx volume
Li et al., 2020 [35]	Retrospective clinical study	22 pts (18 f, 4 m) mean age: 22.6 ± 4.5 yrs range: 18–35 yrs	NO	Young adults (18–35 years old) with transverse maxillary discrepancy; successful opening of the mid-palatal suture; availability of CBCT images obtained before and after expansion.	Maxillary skeletal expander	Nasal cavity volume Nasopharynx volume, Palatopharynx volume Glossopharynx volume Hypopharynx volume
Moschik, 2018 [36]	Retrospective clinical study	16 pts (10 f, 6 m) mean age: 20.7 yrs range: 17–26 yrs	Tooth-borne group: 6 pts (3 f, 3 m) mean age: 12.2 yrs range: 9–15 yrs	Non-growing (CVMS IV) maxillary transverse deficiency; had CBCT imaging done before and after expansion; visible split of mid-palatal suture on CBCT, received no previous orthodontic treatment; had no craniofacial abnormalities	Maxillary skeletal expander	Nasal cavity volume
Kavand et al., 2019 [37]	Retrospective clinical study	18 pts (12 f, 6 m) mean age: 14.7 ± 1.4 yrs range: 11–15 yrs	Tooth-borne group: 18 pts (10 f, 8 m); mean age: 14.4 ± 1.3 yrs range: 11–15 yrs	Individuals between 11 and 15 years of age with no history of orthodontic treatment; temporomandibular joint disorder; adenoidectomy or tonsillectomy; periodontal diseases; systemic diseases; craniofacial anomalies; and no active caries; bilateral maxillary crossbite	Bone-borne rapid maxillary expander	Nasal cavity volume Nasopharynx volume Oropharynx volume Maxillary sinus volume
Atia et al., 2019 [31]	Prospective clinical study	10 pts (all man) range: 12–14 yrs	Conventional hyrax group: 10 pts (all man); range: 12–14 yrs	All patients were males; aged 12 to 14 years old; all patients were free from any syndrome or congenital defects that may affect the craniofacial structures; no previous orthodontic treatment; no previous history of facial or cranial trauma; absence of any breathing disorders; maxillary constriction	Hybrid hyrax expander	Oropharynx volume
Cheung et al., 2021 [42]	Randomized controlled trial	19 pts (11 f, 8 m) mean age: 14.3 ± 1.7 yrs range: 10–16 yrs	Hyrax group and Keles group (random allocation from the total sample as 1:1:1 ratio)	Unilateral or bilateral posterior crossbite; maxillary transverse deficiency of more than 5 mm; erupted first permanent molars and premolars; adequate oral hygiene; and no history of previous orthodontic treatment and no history of craniofacial defects, syndromes, or surgery	Hybrid hyrax expander	Nasal cavity volume Nasopharynx volume Oropharynx volume Hypopharynx volume Maxillary sinus volume
Kim et al., 2021 [32]	Prospective clinical study	26 pts mean age: 13.6 ± 2.9 yrs range: 9–18 yrs	NO	Diagnosed with OSA based on the AHI criteria and maxillary transverse constriction; The patients with syndromic craniofacial deformity, history of orthodontic treatment or adenotonsillectomy, obesity with body mass index (BMI) greater than 23 kg/m^2^, and ATH with Friedman’s classes 3 and 4 were excluded	Maxillary skeletal expander	Nasal cavity volume Nasopharynx volume Palatopharynx volume Glossopharynx volume
Mehta et al., 2021 [38]	Retrospective clinical study	20 pts mean age: 13.69 ± 1.74 years range: 11–15 yrs	Rapid palatal expansion (RPE) group 21 pts, mean age: 13.9 ± 1.14 yrs, and control group 19 pts, mean age: 13.3 ± 1.49 yrs	Patients aged 11 to 15 years, with no history of prior orthodontics, temporomandibular joint disorder, adenoidectomy or tonsillectomy, and the presence of a bilateral maxillary crossbite	Bone-borne rapid maxillary expander	Nasal cavity volume Nasopharynx volume Oropharynx volume Hypopharynx volume
Song, 2020 [39]	Retrospective clinical study	20 pts range: 8–22 yrs	NO	Any age for patients; Using MARPE treatment to correct maxillary transverse discrepancy; No history of previous orthodontic or orthopedic treatment; No history of craniofacial syndrome or deformities	Maxillary skeletal expander	Nasal cavity volume Nasopharynx volume Oropharynx volume
Tang et al., 2021 [40]	Retrospective clinical study	30 pts (21 f, 9 m) mean age: 23.82 ± 3.90 yrs range: 18–33 yrs	NO	Aged >18 years; maxillomandibular skeletal transverse discrepancy 3 mm or greater; no history of expansion treatment or orthognathic surgery; and no severe dentofacial anomalies such as a cleft lip or palate	Maxillary skeletal expander	Nasopharynx volume Oropharynx volume Hypopharynx volume
Hollander, 2021 [41]	Retrospective clinical study	16 pts (12 f, 4 m)	Non-expansion group 8 pts (5 f, 3 m)	Adult patients; maxillary transverse deficiency; successful opening of the mid-palatal suture; non-extraction treatment; and availability of CBCT images; a history of orthodontic treatment and presence of craniofacial syndromes or systemic diseases were excluded	Maxillary skeletal expander	Nasal cavity volume Nasopharynx volume Oropharynx volume

pts, patients; f, female; m, male; yrs, years.

**Table 2:** Expansion and retention protocols of the included studies.

Study	Expansion Device	Expansion Protocol	Duration	Retention
Storto et al., 2019 [30]	Maxillary skeletal expander (supported on U6s, additional skeletal anchorage with four micro-implants)	Twice a day (0.25 mm/turn) until the necessary expansion was achieved	Activated until the complete maxillary expansion	Not reported
Kim et al., 2018 [33]	Modified conventional four-banded hyrax RME appliance (supported on U4s & U6s and additional skeletal anchorage with four micro-implants)	Once a day (0.2 mm/turn) until the required expansion was achieved	The mean duration of expansion was 28 days (range: 18–35 days)	The MARPE appliance was maintained for mean of 15.1 weeks after the completion of the expansion
Yi et al., 2020 [34]	The palatal bracket implant anchorage arch expander (skeletal anchorage with four micro-implants)	Twice a day (0.25 mm/turn) for 14 days until the required expansion was achieved 7 mm	Activated 14 days (expansion was achieved 7mm)	Not reported
Li et al., 2020 [35]	Maxillary skeletal expander (supported on U6s, additional skeletal anchorage with four micro-implants)	Two turns every other day (0.13 mm/ turn) until maxillary skeletal width was no longer less than that of the mandible	The mean duration of expansion was 38 days (range: 30–43 days)	No description of the retention protocol The retention time was at least 3 months
Moschik, 2018 [36]	Maxillary skeletal expander (supported on U6s, additional skeletal anchorage with four micro-implants)	Four times per day, resulting in 0.6 mm activation (0.16mm/turn)	Not reported	Not reported
Kavand et al., 2019 [37]	Bone-borne rapid maxillary expander (skeletal anchorage with two micro-implants)	Twice a day (0.25 mm/turn) until mesio-palatal cusps of the maxillary first molars were in contact with the buccal cusps of mandibular first molars	Activated until the mesio-palatal cusps of the maxillary first permanent molars were in contact with the buccal cusps of mandibular first permanent molars	Not reported
Atia et al., 2019 [31]	Hybrid hyrax (supported on U4s & U6s and additional skeletal anchorage with two micro-implants)	Twice per day for ten days at a constant rate.	Ten consecutive days	Not reported
Cheung et al., 2021 [42]	Hybrid hyrax (supported on U6s and additional skeletal anchorage with two micro-implants)	Twice a day (0.5 mm) until palatal cusps of the upper first molars were in contact with the buccal cusps of the lower first molars	Until palatal cusps of the upper first molars were in contact with the buccal cusps of the lower first molars	The expander was locked, and the patient instructed to return in 6 months
Kim et al., 2021 [32]	Maxillary skeletal expander (supported on U6s and additional skeletal anchorage with four micro-implants)	One turn (0.25 mm) a day for 3–4 weeks	24.3 days (range: 20–26 days)	The expander was removed on 6.2 ± 1.6 months after starting expansion on average
Mehta et al., 2021 [38]	Bone-borne rapid maxillary expander (skeletal anchorage with two micro-implants)	Two turns per day	Not reported	Not reported
Song, 2020 [39]	Maxillary skeletal expander (supported on U4s & U6s and additional skeletal anchorage with four micro-implants)	Depending on the amount of transverse correction needed, the number of turns varied between patients	When the lingual cusps of the maxillary first molars were in edge–edge contact with the buccal cusps of the mandibular first molars, appliance activation was terminated.	Not reported
Tang et al., 2021 [40]	Maxillary skeletal expander (supported on U6s, additional skeletal anchorage with four micro-implants)	Depending on the severity of each patient, ranging from 40–60 turns.	Duration of expansion ranged from 40 to 60 days	The retention after activation was 3 months
Hollander, 2021 [41]	Maxillary skeletal expander (supported on U6s, additional skeletal anchorage with four micro-implants)	Not reported	Not reported	Not reported

U4s, upper first premolars; U6s, upper first molars.

**Table 3:** Measurement method, follow-up points, airway regions, and outcomes of included studies.

Study	Measurement Method	Follow-Up Points	Airway Regions	Treated Group Changes	Change Percentage %
Storto et al., 2019 [30]	CBCT	T0: before expansion T1: immediately after expansion	Nasopharynx volume	T0–T1: 16,058 (2171.98); 21,835.55 (1937.64)	26%
Kim et al., 2018 [33]	CBCT	T0: before expansion T1: immediately after expansion	Nasal cavity volume Nasopharynx volume	ΔT1–T0: 1061.6 (613.9) ΔT1–T0: 513.3 (727.8)	9.9% 6.4%
Yi et al., 2020 [34]	CBCT	T0: before expansion T2: three months after expansion	Nasopharynx volume Oropharynx volume Palatopharynx volume Glossopharynx volume	T0–T2: 5922.61 (1938.28); 6424.61 (1798.58) T0–T2: 21,057.11 (9371.71); 19,972.03 (8026.73) T0–T2: 11,201.39 (4071.85); 11,802.42 (4322.75) T0–T2: 10,020.89 (6403.14); 8527.69 (4679.10)	8.48% N/A N/A N/A
Li et al., 2020 [35]	CBCT	T0: before expansion T2: three months after expansion	Nasal cavity volume Nasopharynx volume Palatopharynx volume Glossopharynx volume Hypopharynx volume	T0–T2: 18,110.7 (6236.8); 21,036.5 (4777.8) T0–T2: 5212.1 (1509.9); 5947.1 (2101.6) T0–T2: 7477.8 (2901.6); 7903.9 (3001.9) T0–T2: 4080.1 (1656.4); 4539.5 (2129.2) T0–T2: 10,597.7 (3925.2); 9373.5 (3576.4)	16.2% 14.1%. 5.7% 11.26% −11.6%
Moschik, 2018 [36]	CBCT	T0: before expansion T1: immediately after expansion	Left nasal cavity volume Right nasal cavity volume Total nasal cavity volume	T0–T1: 10,481.00 (463.4996); 13,695.00 (477.159) T0–T1: 9938.06 (449.1738); 12,730.69 (470.5434) T0–T1: 20,419.06; 26,425.69	N/A N/A 22.73%
Kavand et al., 2019 [37]	CBCT	T0: before expansion T2: three months after expansion	Nasal cavity volume Nasopharynx volume Oropharynx volume Left maxillary sinus volume Right maxillary sinus volume	T0–T2: 14,860 (3109); 16,726 (3041) T0–T2: 3760 (1630); 4580 (1819) T0–T2: 11,746 (4269); 12,297 (3660) T0–T2: 13,004 (3926); 13,739 (3759) T0–T2: 12,369 (4039); 13,184 (3821)	16.1% 20.0% 2.6% 2.1% 5.2%
Atia et al., 2019 [31]	CT	T0: before expansion T1: immediately after expansion	Oropharynx volume 1 Oropharynx volume 2	T0–T1: 13.86 (0.60); 16.82 (0.87) T0–T1: 11.44 (0.28); 13.96 (1.02)	N/A N/A
Cheung et al., 2021 [42]	CBCT	T0: before expansion T3: six months after expansion	Nasal cavity volume Nasopharynx volume Oropharynx volume Hypopharynx volume Maxillary sinus volume	T0–T3: 26,630.8 (5659.0); 29,319.5 (5536.7) T0–T3: 5416.8 (2194.0); 6362.4 (2443.8) T0–T3: 11,651.8 (6208.3); 12,702.7 (5678.1) T0–T3: 3441.9 (1430.0); 3451.3 (1290.9) T0–T3: 23,433.05 (9577.7); 23,813.4 (8131.1)	10.1% 17.5% 9.0% 0.3% 10.0%
Kim et al., 2021 [32]	CBCT	T0: before expansion T3: six months after expansion	Nasal cavity volume Nasopharynx volume Palatopharynx volume Glossopharynx volume	T0–T3: 22,987.80 (9483.35); 40,755.54 (13,083.33) T0–T3: 5072.68 (1533.46); 7502.24 (2049.73) T0–T3: 9060.23 (4072.48); 11,236.53 (4404.78) T0–T3: 9861.37 (3464.25); 12,122.51 (3727.92)	77.2% 47.9% 24.0%. N/A
Mehta et al., 2021 [38]	CBCT	T0: before expansion T3: six months after expansion	Nasal cavity volume Nasopharynx volume Oropharynx volume Hypopharynx volume	T0–T3: 16,204.1 (3100.53); 18,475.95 (3329.13) T0–T3: 3412.89 (1425.84); 4158.32 (1459.81) T0–T3: 6270.35 (2617.56); 7675.74 (3047.01) T0–T3: 6662.93 (3459.65); 8361.92 (3321.25)	14.4% 21.8% 19.2% 4.4%
Song, 2020 [39]	CBCT	T0: before expansion T1: immediately after expansion	Nasal cavity volume Nasopharynx volume Oropharynx volume	T0–T1:15,892.7 (3025.0); 18,131 (4814.1) T0–T1: 5874.6 (2172.6); 4879.2 (1847.6) T0–T1: 17,855.4 (7806.0); 15,914.2 (7137.3)	N/A
Tang et al., 2021 [40]	CBCT	T0: before expansion T2: three months after expansion	Nasopharynx volume Oropharynx volume Hypopharynx volume	T0–T2: 6463.86 (1459.17); 7806.69 (1806.87) T0–T2: 10,886.67 (3382.94); 11,849.28 (4306.25) T0–T2: 8542.31 (3426.18); 8307.14 (3237.12)	N/A
Hollander, 2021 [41]	CBCT	T0: before expansion T1: immediately after expansion	Nasal cavity volume Nasopharynx volume Oropharynx volume	T0–T1: 80,448.93 (15,387.18); 87,446.73 (15,345.97) T0–T1: 8572.62 (3354.84); 10,191.66 (3808.14) T0–T1: 8624.04 (4758.53); 12,505.92 (6336.88)	9.21% 19.99% 54.88%

N/A: information not available.

## Data Availability

Not applicable.

## Supplementary Materials

The following supporting information can be downloaded at: https://www.mdpi.com/article/10.3390/jcm12051790/s1, Table S1: Electronic databases used and search strategy; Table S2: Search results by database; Table S3: Studies excluded at full-text review with reasons; Table S4: Upper airway boundary of the included studies in this systematic review [30,31,32,33,34,35,36,37,38,39,40,41,42]; Table S5: The main in-formation of the CBCT or CT analysis in this systematic review [30,31,32,33,34,35,36,37,38,39,40,41,42]; Table S6: Summary of findings table according to the GRADE approach; Table S7: Results of meta-analysis of all includ-ed studies; Table S8: Egger’s test was used to test the publication bias of the included studies; Table S9: Sensitivity analysis for the changes of upper airway volume after expansion and retention [32,34,35,37,38,39,40,41,42]; Figure S1: Results of the risk of bias assessment in the individual studies the Re-vised Cochrane Risk of Bias Tool for randomized trials (ROB2) [42].
