# Supplementary material for: Three-Dimensional Evaluation Effects of Microimplant-Assisted Rapid Palatal Expansion on the Upper Airway Volume: A Systematic Review and Meta-Analysis
Source: J Clin Med. 2023 Feb 23;12(5):1790. doi: 10.3390/jcm12051790 (PMC10003187; doi:10.3390/jcm12051790)
Supplement: Supplementary file 1 [file jcm-12-01790-s001.zip › jcm-2146825-supplementary.pdf]

Table S1. Electronic databases used and search strategy

Database	Search strategy
Medline via Ovid	(1) rapid maxillary expan$. ab, ti; (2) rapid palatal expan$. ab, ti; (3) maxillary expan$. ab, ti; (4) skeletal expan$. ab, ti; (5) palatal expan$. ab, ti; (6) rapid maxillary disjunction. ab, ti; (7) rapid palatal disjunction. ab, ti; (8) MARME. ab, ti; (9) MARPE. ab, ti; (10) #1 or #2 or #3 or #4 or #5 or #6 or #7 or #8 or #9; (11) air$. ab, ti; (12) volum$. ab, ti; (13) #11 or #12; (14) #10 and #13
Embase	(1) 'maxillary'/exp or maxillary; (2) palatal; (3) skeletal; (4) expan* (5) 'disjunction'/exp or disjunction; (6) #1 and #4; (8) #2 and #4; (9) #3 and #4; (10) #1and #5; (11) #2and #5; (12) #6 or #7 or #8 or #9 or #10 or #11; (13) air*; (14) volum*; (15) #13 or #14; (16) #12 and #15 and [embase]/lim and [humans]/lim and [clinical study]/lim
Scopus	all (MARPE or MARME or rapid maxillary expansion or rapid palatal expansion or rapid maxillary disjunction or rapid palatal disjunction) and all (air* or volum*)
Web of Science	(1) TS = (MARME OR MARPE OR rapid palatal expansion OR rapid maxillary expansion OR rapid palatal disjunction OR rapid maxillary disjunction); (2) TS = (volum* OR air*); (3) # (1) AND # (2)
Cochrane Library	(1) maxillary: ti, ab, kw; (2) expan*: ti, ab, kw; (3) palatal: ti, ab, kw; (4) #1 and #2; (5) #2 and #3 (6) #4 or #5; (7) air*: ti, ab, kw; (8) volum*: ti, ab, kw; (9) #7 or #8; (9) #6 and #9
ProQuest	AB, TI (MARPE or MARME or maxillary expansion or palatal expansion or maxillary disjunction or palatal disjunction)

Table S2. Search results by database

**Database**	**Results**
Medline via Ovid	345
Embase	464
Scopus	106
Web of science	459
Cochrane Library	80
ProQuest	195
Total	1649

Table S3. Studies excluded at full-text review with reasons

Article title	Reason excluded
Exploratory work on the effect of rapid maxillary expansion on nasal airway dimensions	Not use CBCT or CT to measure the upper airway volume
Nasal airway changes in bone-borne and tooth-borne rapid maxillary expansion treatments	
Rapid-maxillary-expansion induced rhinological effects: a retrospective multicenter study	Not report the upper airway of the MARPE group individually
A randomized control trial investigating the effectiveness of three different upper jaw expansion devices in correcting narrow upper jaws in 10 to16 year old children	The studies are ongoing
Evaluation of changes produced by 2 types of appliances to correct the narrow upper jaw	
Comparing and evaluating upper airway changes between conventional palatal expansion and palatal expansion with implants using CBCT	
Dentoskeletal and upper airway changes after micro-implant-assisted palatal expansion (MARPE) in patients in the final stages of suture fusion: a randomized controlled clinical trial	
Mini-implant assisted rapid palatal expansion (MARPE) effects on adult obstructive sleep apnea (OSA) and quality of life: a multi-center prospective controlled trial	No upper airway volume assessment

Table S4. Upper airway boundary of the included studies in this systematic review

Study	Measure region	Upper airway boundary			
		**Anterior boundary**	**Posterior boundary**	**Superior boundary**	**Inferior boundary**
Cheung et al., 2021 [42]	Nasal cavity	ANS to N	S to PNS	N to S	ANS to PNS
	Nasopharynx	S-PNS	S to the tip of the odontoid process		PNS to the tip of the odontoid process
	Oropharynx	PNS to the epiglottis	Tip of the odontoid process to the posterior-superior border of the CV4	PNS to the tip of the odontoid process	The base of the epiglottis to the posteriorsuperior border of the CV4
	Hypopharynx	The base of the epiglottis to the inferior border of the symphysis	The posteriorsuperior corner of the CV4 to the posterior-inferior corner of the CV4	The base of the epiglottis to the posterior-superior corner of the CV4	The posterior-inferior corner of the CV4 to the inferior border of the symphysis
Kavand et al., 2019 [37]	Nasal cavity	ANS to N	S to PNS	N to S	

	Nasopharynx	S-PNS	S to the tip of odontoid process		PNS to the tip of the odontoid process
	Oropharynx		The tip of odontoid process to the most anterior-inferior point of CV3	PNS to the tip of odontoid process	The most anterior–inferior point of CV3 to menton
Kim et al., 2021 [32]	Nasal cavity	Na-per plane	Se-PNS plane	SN plane	
	Nasopharynx	Se-PNS plane		SN plane	PNS plane
	Palatopharynx			PNS plane	SPT plane
	Glossopharynx			SPT plane	Eb plane
Kim et al., 2018 [33]	Nasal cavity	ANS-perp. plane	Choanae plane	FH plane	
	Nasopharynx			choanae plane	C3 plane
Li et al., 2020 [35]	Nasal cavity	ANS to N	S to PNS	N to S	
	Nasopharynx	S-PNS	Pharyngeal posterior wall		The line parallel to (FHP) passing through PNS point
	Palatopharynx	Pharyngeal anterior wall	Pharyngeal posterior wall		The line parallel to FHP passing through the tip of the uvula
	Glossopharynx	Pharyngeal anterior wall	Pharyngeal posterior wall		The line parallel to the FHP passing through the top of the

					epiglottis
	Hypopharynx	Pharyngeal anterior wall	Pharyngeal posterior wall		The line parallel to the FHP passing through the anterior–inferior point of CV4
Mehta et al., 2021 [38]	Nasal cavity	ANS to N	S to PNS	N to S	ANS to PNS
	Nasopharynx	PNS	The tip of odontoid process	Sella	The PNS to tip of odontoid process
	Oropharynx	PNS to the point parallel to CV2	Tip of odontoid process and CV2	PNS to tip of odontoid process	The line connecting the CV2 parallel to HRF to the anterior boundary
	Hypopharynx	PNS to point parallel to CV4	Anteroinferior point of CV2 and CV4	The inferior border of oropharynx	The line joining CV4 to anterior boundary parallel to HRF
Song, 2020 [39]	Nasal cavity	ANS to N	S to PNS	N to S	ANS to PNS

	Nasopharynx	S-PNS	S to the tip of the odontoid process		PNS to the tip of the odontoid process
	Oropharynx		The tip of odontoid process to the most anterior-inferior point of CV3	PNS to the tip of odontoid process	Menton to the most anterior-inferior point of the third cervical vertebrae
Storto et al., 2019 [30]	Nasopharynx			Choanae plane	C3 plane
Tang et al., 2021 [40]	Nasopharynx	S-PNS	Pharyngeal posterior wall		The line parallel to the FHP passing through PNS
	Oropharynx		Pharyngeal posterior wall	The line parallel to the FHP passing through PNS	The line parallel to the FHP passing through the top of the epiglottis
	Hypopharynx		Pharyngeal posterior wall	The line parallel to the FHP passing through the top of the epiglottis	The line parallel to the FHP passing through the anterior and inferior point of CV4
Yi et al., 2020	Nasopharynx				

[34]		S-PNS			PNS plane
	Palatopharynx			PNS plane	SP plane
	Glossopharynx			SP plane	C3pi plane
	Oropharynx			PNS plane	C3pi plane
Atia et al., 2019 [31]	Oropharynx	The level of the hard and soft palate to the level of the lower border of the uvula			
Hollander, 2021 [41]	Nasal cavity	ANS to N	S to PNS	N to S	ANS to PNS
	Nasopharynx	S-PNS	S to the tip of the odontoid process	S to the tip of the odontoid process	PNS to the tip of the odontoid process
	Oropharynx	PNS to the epiglottis	Tip of the odontoid process to the posterior-superior border of CV4	PNS to the tip of the odontoid process	The base of the epiglottis to the posteriorsuperior border of the CV4
Moschik, 2018 [36]	Nasal cavity	ANS to N			ANS to PNS, laterally, the region of interest extended into the nasal

					sinus, enclosing the whole walls of the nasal cavity.

N, nasion. ANS, anterior nasal spine. S, sella. PNS, posterior nasal spine. CV2, cervical vertebrae 2. CV4, cervical vertebrae 4. CV3, cervical vertebra 3. Choanae, the choanae are bounded medially by the vomer, inferiorly by the horizontal plate of the palatine bone, laterally by the medial pterygoid plate, and superiorly by the body of the sphenoid bone. C3, the most inferior and anterior point on the third cervical vertebra. FH plane, Frankfort horizontal plane, which is determined by both porions and left orbitale. SPT, tip of soft palate. Eb, epiglottis base. C3 plane, Parallel to the FH plane and passing through C3. ANS-perp. Plane, perpendicular to the FH plane and passing through ANS. Choanae plane, the plane along the choanae. Na-per plane, the plane perpendicular to the FH plane and the MSP passing through Na. MSP, midsagittal plane perpendicular to the FH plane passing through ANS and PNS. Palatal plane, the plane perpendicular to the MSP passing through ANS and PNS. SPT plane, the plane parallel to the FH plane passing through SPT. FHP, Frankfort horizontal plane.

Table S5. The main information of the CBCT or CT analysis in this systematic review

Study	3D imaging	Scanning setting parameters	Analysis software	Patient position	
Cheung et al., 2021 [42]	CBCT (NewTom 5G, Cone Beam 3D Imaging, Verona, Italy)	110 kV, 20 mA<br>FOV: 18 × 16 cm<br>Voxel size: 0.3 mm<br>3.6 seconds per section	Dolphin Imaging software (Dolphin Imaging, Chatsworth, California, USA)	Supine position	Patients were instructed after expiration to be in centric occlusion with the lips and tongue in resting position and not to swallow
Kavand et al., 2019 [37]	CBCT (iCAT, Imaging Sciences International, Hartfield, PA)	120 kV, 20 mA<br>Voxel size: 0.3mm<br>Scan time: 8.9 s	Dolphin Imaging Software, version 11.0 (Dolphin Imaging, Chatsworth, CA)		
Kim et al., 2021 [32]	CBCT	80 kV, 2 mA<br>FOV: 15.4 × 15.4 cm<br>Voxel size: 0.3 mm<br>Exposure time: 17 s	The Invivo software (Anatomage version 5.0, San Jose, CA)<br>Mimics software (Materialise NV version 20.0, Leuven, Belgium)<br>3-Matic software	Upright position	Holding their breath at the end of expiration when static and consistent pharyngeal airway dimensions were recorded during a respiratory cycle
Kim et al., 2018	CBCT (Alphard VEGA,	120 kV, 8 mA	OnDemand3D software		

[33]	ASAHI Roentgen IND, Kyoto, Japan)	FOV: 100 × 100 mm² or 154x154 mm²<br>Voxel size: 0.3 mm<br>Captured for 17 s	(Cybermed Co., Seoul, Korea)		
Li et al., 2020 [35]	CBCT (Quantitative Radiology, Verona, Italy)	110 kV, 7.33 mA<br>FOV: 18 × 16 cm<br>Voxel size: 0.3 mm<br>Emission time: 4.8 s	Dolphin Imaging software (Chatsworth, CA, USA)	Supine position	Keeping the teeth in centric occlusion and the tongue in the position at the end of swallowing (against the palate), breathing smoothly, and no swallowing
Mehta et al., 2021 [38]	CBCT (iCAT Imaging Sciences International, Hartfield, Penn)	120 kV, 20 mA<br>Voxel size 0.3 mm<br>Scan time: 8.9 s	Dolphin Imaging software (Version 11.9; Dolphin Imaging and Management Solutions, Chatsworth, Calif)		
Song, 2020 [39]	CBCT (Gendex GX-DP-700 or New Tom VGi 9 cone beam, Imola, Italy).	85 kV, 4.0 mA<br>Scan time: 11.30 s<br>FOV: 17 × 13 cm<br>300 voxel size and 16-bit gray scale	Dolphin Imaging software software (version 11.95)	Supine position	
Storto et al., 2019 [30]	CBCT (iCATScanner machine (Imaging Sciences International, Hatfield, PA)	120 kV, 36 mAs<br>FOV: 16x 13 cm<br>Voxel size: 0.25 mm	OnDemand3D software (Cybermed, Seoul, South Korea)		
Tang et al., 2021	CBCT (NewTom 5G,	110 kV, 7.33 mA	Dolphin Imaging software	Supine	Keeping the teeth in centric

[40]	Verona, Italy)	FOV: 18 × 16 cm<br>Voxel: 0.3 mm<br>Scan time, 4.8 s	(version 11.8; Dolphin Imaging and Management Solutions, Chatsworth, Calif).	position	occlusion, the tongue in the position at the end of swallowing (against the palate), breathing smoothly, no swallowing
Yi et al., 2020 [34]	CBCT (ProMax 3D, MaxPLANMECA Oy, Helsinki, Finland)	96 kV, 10 mA<br>Captured for 12 s	Dolphin Imaging software (Version 11.9 Solutions, USA)	Upright position	Occluding in the centric occlusion, breathe smoothly and not to swallow
Hollander, 2021 [41]	CBCT (NewTom 5G)	110 kV<br>FOV: 18 × 16 cm<br>Voxel: 0.3 mm<br>Scan times: 18 s (3.6 s emission time)	AMIRA software		
Atia et al., 2019 [31]	CT		Mimics software		
Moschik, 2018 [36]	CBCT (NewTom 5G)	110 kV<br>FOV: 18 × 16 cm<br>Scan times: 18 s (3.6 s emission time)	OnDemand3D software (version 1.0.10.5385; Cybermed, Seoul, Korea)		

CT, Computer tomography; CBCT, Cone beam computer tomography

Table S6. Summary of findings table according to the GRADE approach

Quality assessment							Summary of findings		
**No of studies**	**Design**	**Risk of bias**	**Inconsistency**	**Indirectness**	**Impression**	**Publication bias**	**No of patients**	**Difference in means (95% CI)**	**Quality**
**Nasal cavity: post- expansion**									
2	Observational studies	Serious	Not serious (I^2 = 0.0%, p = 0.396)	Not serious	Not serious	N/A	33	2527.23mm^3 [-92.53,5147.00]	⊕⊕⊕⊖ MODERATE
**Nasal cavity: post- retention (follow-up: range 3-6 months)**									
5	4 observational studies 1 randomized controlled trial	Serious	Serious (I^2 = 67.6%, p = 0.015)	Not serious	Not serious	Not serious (p = 0.849)	93	3646.27mm^3 [1082.77,6209.77]	⊕⊕⊖⊖ LOW
**Nasopharynx: post-expansion**									
2	Observational studies	Serious	Not serious (I^2 = 0.0%, p = 0.666)	Not serious	Not serious	N/A	33	1138.29mm^3 [-52.04,2328.61]	⊕⊕⊕⊖ MODERATE

**Nasopharynx: post-retention (follow-up: range 3-6 months)**									
7	6 observational studies 1 randomized controlled trial	Serious	Not serious (I^2 = 7.8%, p = 0.369)	Not serious	Not serious	Not serious (p = 0.756)	142	1021.10mm^3 [597.11,1445.08]	⊕⊕⊕⊖ MODERATE
**Oropharynx: post-expansion**									
2	Observational studies	Serious	Not serious (I^2 = 0.0%, p = 0.549)	Not serious	Not serious	N/A	33	3156.84mm^3 [83.63,6230.06]	⊕⊕⊕⊖ MODERATE
**Oropharynx: post-retention (follow-up: range 3-6 months)**									
5	4 observational studies 1 randomized controlled trial	Serious	Not serious (I^2 = 0.0%, p = 0.853)	Not serious	Not serious	Not serious (p = 0.628)	106	789.26mm^3 [-171.25,1749.76]	⊕⊕⊕⊖ MODERATE

N/A: information not available

Table S7. Results of meta-analysis of all included studies

Outcome		Time point	Number of studies	WMD (mm³)	95% CI	p value	I²
Nasal cavity volume		T1	2	2527.233	-92.530, 5146.996	0.059	0.0%
	Post-retention	T2	2	2632.577	595.496, 4669.658	0.011*	0.0%
		T3	3	5804.864	207.289 ,1. 1e+04	0.042*	83.7%
	Post-retention	Overall: T2 and T3	5	3646.274	1082.774, 6209.775	0.005*	67.6%
Nasopharynx volume		T1	2	1138.287	-52.035, 2328.610	0.061	0.0%
	Post-retention	T2	4	915.100	404.917, 1425.284	0.000*	0.0%
		T3	3	1311.208	275.635, 2346.781	0.013*	53.9%
	Post-retention	Overall: T2 and T3	7	1021.096	597.112, 1445.080	0.000*	7.8%
Oropharynx volume		T1	2	3156.843	83.629, 6230.056	0.044*	0.0%
	Post-retention	T2	3	475.281	-727.364, 1677.926	0.439	0.0%
		T3	2	1342.287	-253.822, 2938.395	0.099	0.0%
	Post-retention	Overall: T2 and T3	5	789.258	-171.248, 1749.764	0.107	0.0%

Palatopharynx volume		T1	0	0	0	0	0
	Post-retention	T2	2	465.642	-1.1e+03, 2000.443	0.552	0.0%
		T3	1	2176.300	-966.075, 5318.675	0.175	N/A
	Post-retention	Overall: T2 and T3	3	795.127	-583.968, 2174.223	0.258	0.0%
Glossopharynx volume		T1	0	0	0	0	0
	Post-retention	T2	2	-525.558	-1.6e+03, 565.019	0.345	0.0%
		T3	1	2261.140	-404.618, 4926.897	0.096	N/A
	Post-retention	Overall: T2 and T3	3	184.496	-1.7e+03, 2114.957	0.851	47.4%
Hypopharynx volume		T1	0	0	0	0	N/A
	Post-retention	T2	2	-597.362	1.9e+03, 745.432	0.383	0.0%
		T3	2	571.682	-988.755, 2132.120	0.473	52.9%
	Post-retention	Overall: T2 and T3	4	39.846	-809.768, 889.461	0.927	19.9%

WMD: weighted mean difference; CI: confidence interval; N/A: information not available; T1: immediately after expansion, T2: three months after expansion, T3: six months after expansion; *Statistically significant.

Table S8. Egger's test was used to test the publication bias of the included studies

**Outcome**	**Time point**	**Number of studies**	**Publication bias (p value)**
Nasal cavity volume	post-expansion	2	N/A
	post-retention	5	p = 0.849
Nasopharynx volume	post-expansion	2	N/A
	post-retention	7	p = 0.756
Oropharynx volume	post-expansion	2	N/A
	post-retention	5	p = 0.628
Hypopharynx volume	post-expansion	0	N/A
	post-retention	4	p = 0.942
Palatopharynx volume	post-expansion	0	N/A
	post-retention	3	p = 0.528
Glossopharynx volume	post-expansion	0	N/A
	post-retention	3	p = 0.784

N/A: information not available

Table S9. Sensitivity analysis for the changes of upper airway volume after expansion and retention.

**Study omitted**	**Estimate**	**95% CI**
Nasal cavity: post-expansion		
Hollander et al. 2021 [41]	2239.2002	-463.63922, 4942.0396
Song et al. 2020 [39]	6997.7969	-3650.51, 17646.104
Combined	2527.2328	-92.529892, 5146.9955
Nasal cavity: post-retention		
Li et al. 2020 [35]	4142.7271	786.51825, 7498.9355
Kim et al. 2021 [32]	2481.5308	1158.6801, 3804.3813
Kavand et al. 2019 [37]	4451.3916	837.50018, 8065.2827
Cheung et al. 2021 [42]	4153.5415	875.13757, 7431.9458
Mehta et al. 2021 [38]	4628.0752	841.6203, 8414.5303
Combined	3646.2742	1082.7736, 6209.7748
Nasopharynx: post-expansion		
Hollander et al. 2021 [41]	995.3999	-360.32266, 2351.1226
Song et al. 2020 [39]	1619.04	-867.72491, 4105.8052
Combined	1138.2873	-52.035294, 2328.6099
Nasopharynx: post-retention		
Yi et al. 2020 [34]	1092.5858	629.30475, 1555.8668
Tang et al. 2021 [40]	931.31494	430.84259, 1431.7874
Li et al. 2020 [35]	1071.0027	577.24341, 1564.7618
Kim et al. 2021 [32]	879.4021	454.95563, 1303.8485
Kavand et al. 2019 [37]	1074.1006	583.83069, 1564.3705
Cheung et al. 2021 [42]	1030.3921	545.03937, 1515.7448
Mehta et al. 2021 [38]	1088.991	583.14233, 1594.8395
Combined	1021.0959	597.11164, 1445.0802
Oropharynx: post-expansion		
Hollander 2021 [41]	1941.2002	-3086.7334, 6969.1338
Song et al. 2020 [39]	3881.8799	-1.1141176, 7764.874
Combined	3156.8428	83.629219, 6230.0563
Oropharynx: post-retention		
Yi et al. 2020 [34]	847.16632	-128.06477, 1822.3975
Tang et al. 2021 [40]	734.43762	-367.52542, 1836.4006
Kavand et al. 2019 [37]	1082.9955	-124.86248, 2290.8535
Cheung et al. 2021 [42]	771.22882	-221.81912, 1764.2767
Mehta et al. 2021 [38]	528.11584	-618.00647, 1674.2382
Combined	789.25784	-171.2485, 1749.7642
Palatopharynx: post-retention		
Li et al. 2020 [35]	1409.8741	-841.83807, 3661.5864
Yi et al. 2019 [34]	838.45978	-686.82971, 2363.7493

Kim et al. 2021 [32]	465.64206	-1069.1589, 2000.443
Combined	795.12738	-583.96844, 2174.2232
Glossopharynx: post-retention		
Li et al. 2020 [35]	781.9267	-2813.6301, 4377.4834
Yi et al. 2019 [34]	621.67761	-1987.6415, 3230.9968
Kim et al. 2021 [32]	-525.55841	-1616.1357, 565.01892
Combined	184.49563	-1745.9657, 2114.957
Hypopharynx: post-retention		
Tang et al. 2021 [40]	140.89519	-1137.9957, 1419.7861
Cheung et al. 2021 [42]	79.903221	-1490.726, 1650.5323
Li et al. 2020 [35]	211.33708	-647.70837, 1070.3824
Mehta et al. 2021 [38]	-168.90385	-896.81885, 559.01111
Combined	39.846064	-809.76844, 889.46057

CI: confidence interval

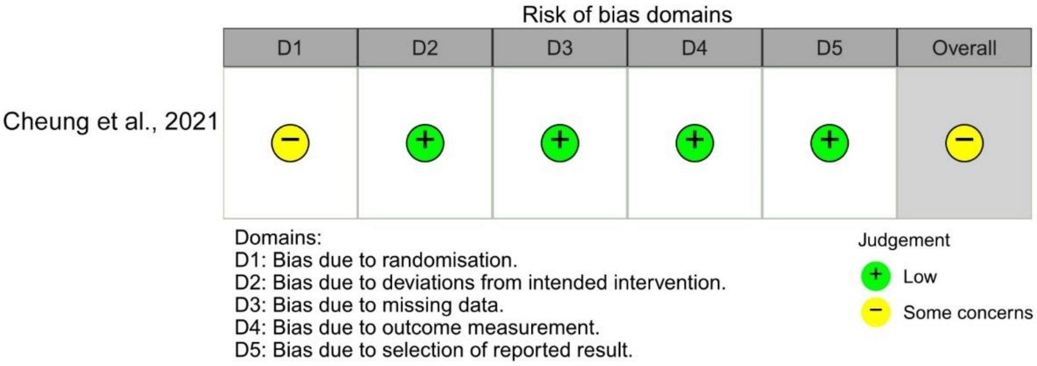

Figure S1. Results of the risk of bias assessment in the individual studies the Revised Cochrane Risk of Bias Tool for randomized trials (ROB2).
